# Supplementary material for: The evolution of sexual signaling is linked to odorant receptor tuning in perfume-collecting orchid bees
Source: Nat Commun. 2020 Jan 13;11:244. doi: 10.1038/s41467-019-14162-6 (PMC6957680; doi:10.1038/s41467-019-14162-6)
Supplement: Supplementary file 1 — Supplementary Information [file 41467_2019_14162_MOESM1_ESM.pdf]

Supplementary Information

The evolution of sexual signaling is linked to odorant receptor tuning in perfume-collecting orchid bees

Brand et al.

Table of contents

Supplementary Figures 2
Supplementary Tables 11
Supplementary Discussion 20
Supplementary References 20

Supplementary Figures

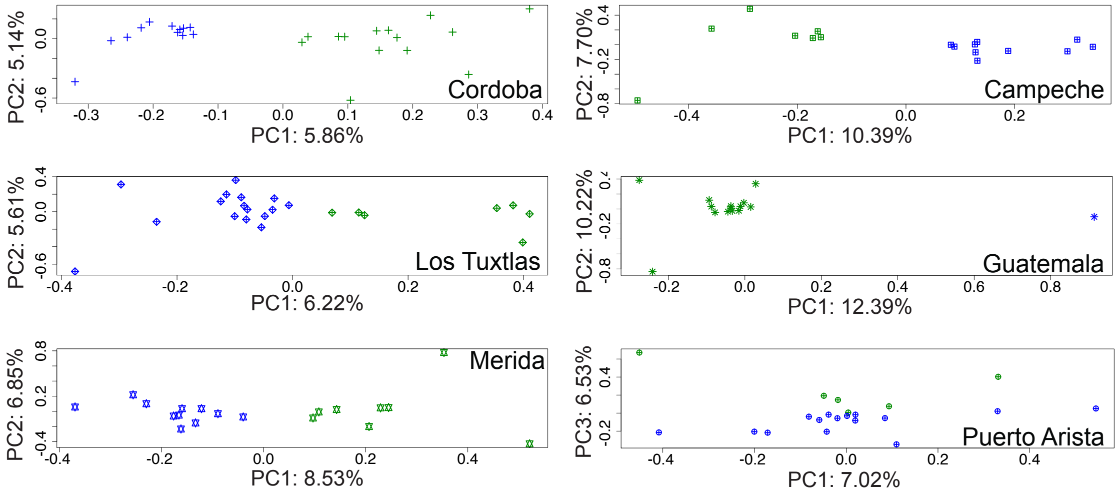

Supplementary Figure 1 PCAs of sampling sites where *E. dilemma* (green) and *E. viridissima* (blue) co-occur show that individuals cluster by species, suggesting genetic differentiation.

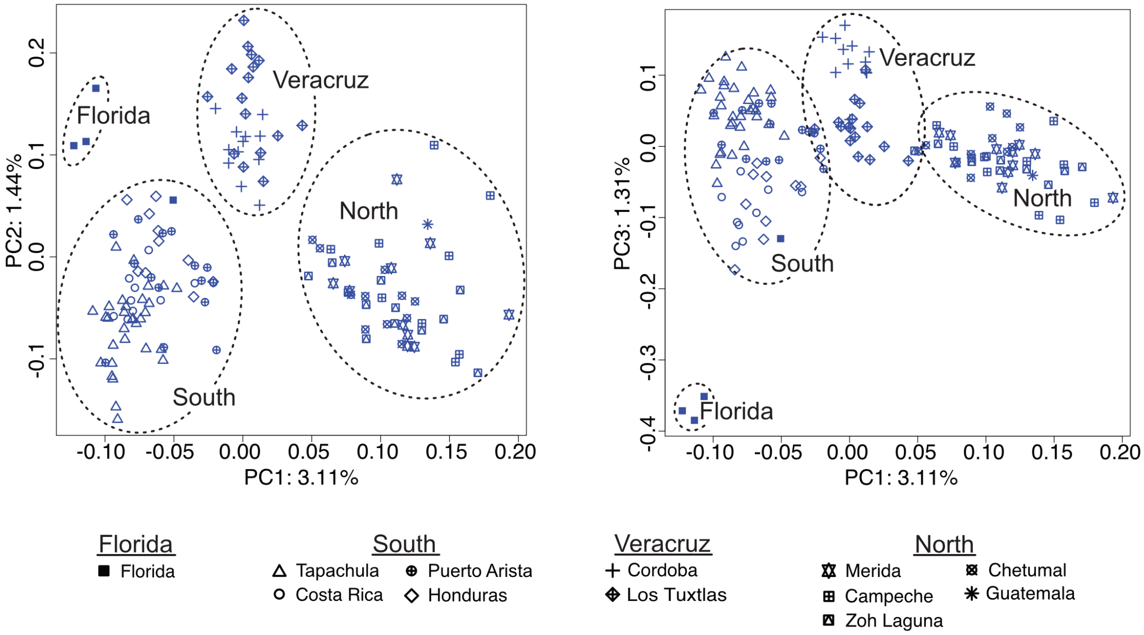

Supplementary Figure 2 Population structure within *E. dilemma*. The first three PC axes of a PCA based on GBS data of all *E. dilemma* individuals analyzed reveal population structure roughly corresponding to geography and allopatry – sympatry with *E. dilemma*. The first PC explains about 3.11% of genetic variation, about twice as much as either PC2 or PC3. Geographic regions are indicated. These results indicate that the southern *E. dilemma* population (Ed_{south}) might be the source for the recently introduced Florida population.

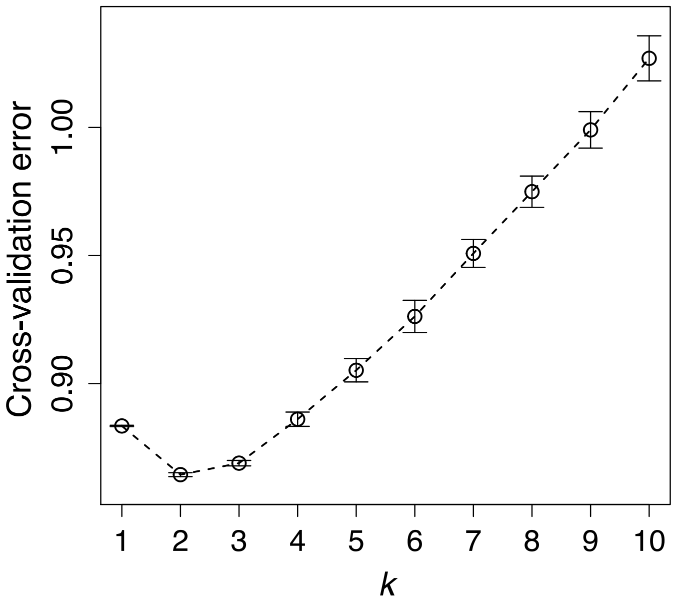

k	CV error
1	0.883
2	0.864
3	0.869
4	0.886
5	0.905
6	0.926
7	0.951
8	0.975
9	0.999
10	1.027

Supplementary Figure 3 ADMIXTURE mean cross-validation (CV) errors for k of 1 through 10. Lower cross validation errors indicate higher support for a given number of populations (k) for the underlying data. k=2 and 3 have the most favorable CV errors with a differential of less than 0.005. Error bars in the plot represent the standard deviation of the mean.

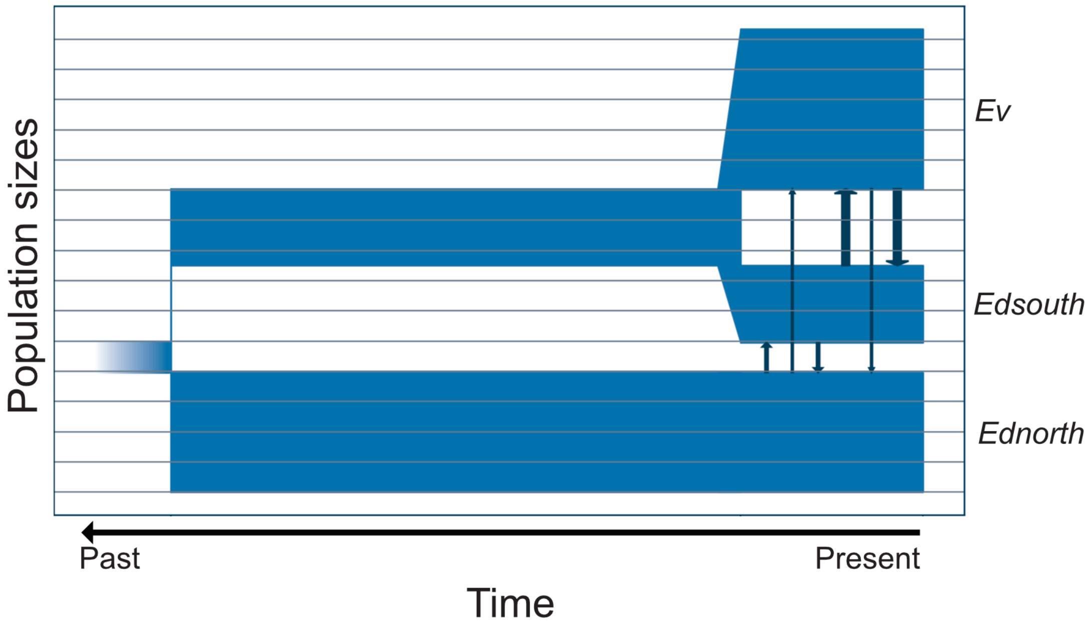

Supplementary Figure 4 Preferred demography model. The highly preferred demography model (AIC weight = 1) suggests that *E. viridissima* evolved from within *E. dilemma*. In this model Ed_{north} and Ed_{south} split first followed by a split of *Ev* from Ed_{south} (Supplementary Table 6). Arrows between the three lineages indicate migration vertices.

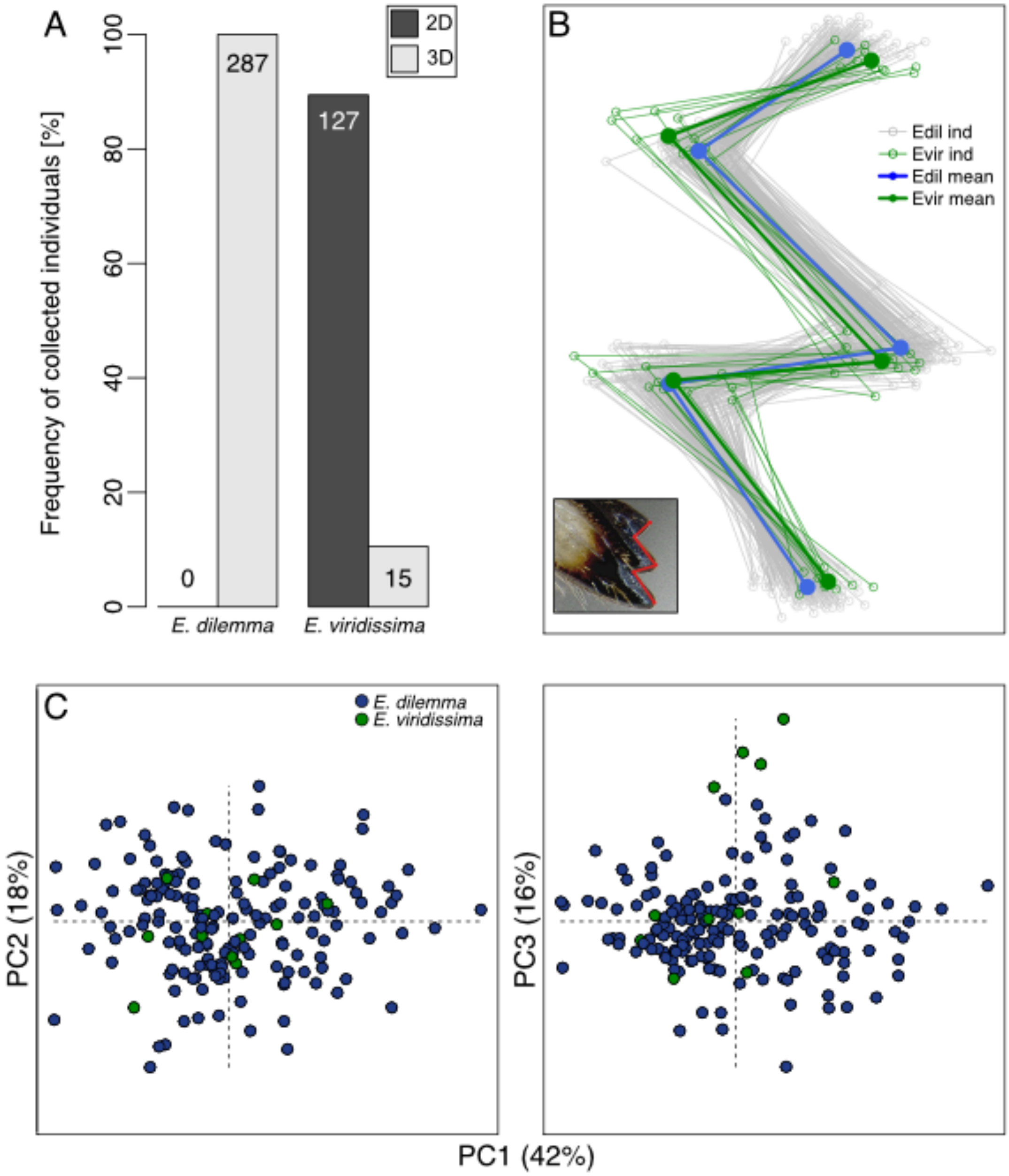

Supplementary Figure 5 Geometric morphological analysis of tridentate mandibles. A) While all *E. dilemma* males collected were tridentate (3D, light grey), *E. viridissima* was polymorphic for the number of mandibular teeth with 10.6% of the analyzed males revealing tridentate and 89.4% bidentate (2D, dark grey) mandibles, all of which were collected in sympatric populations. **B)** Individual landmarks of 175 tridentate mandibles were aligned for *E. dilemma* (N = 164, grey) and *E. viridissima* (N = 11, green) individuals (above left). Mean landmarks are indicated in thick lines and solid points for *E. dilemma* (green) and *E. viridissima* (blue) and suggest that overall the middle tooth in *E. viridissima* is shifted towards the basal tooth. The inlet indicates how landmarks were set on stacked photos of mandibles. **C)** PCA of mandible shape reveals no species-specific clustering of *E. dilemma* (blue) and *E. viridissima* (green) over the first two PC axes (left). The third PC axis reveals clustering of four of the 11 *E. viridissima* individuals. Variance explained by the respective axes is indicated in brackets.

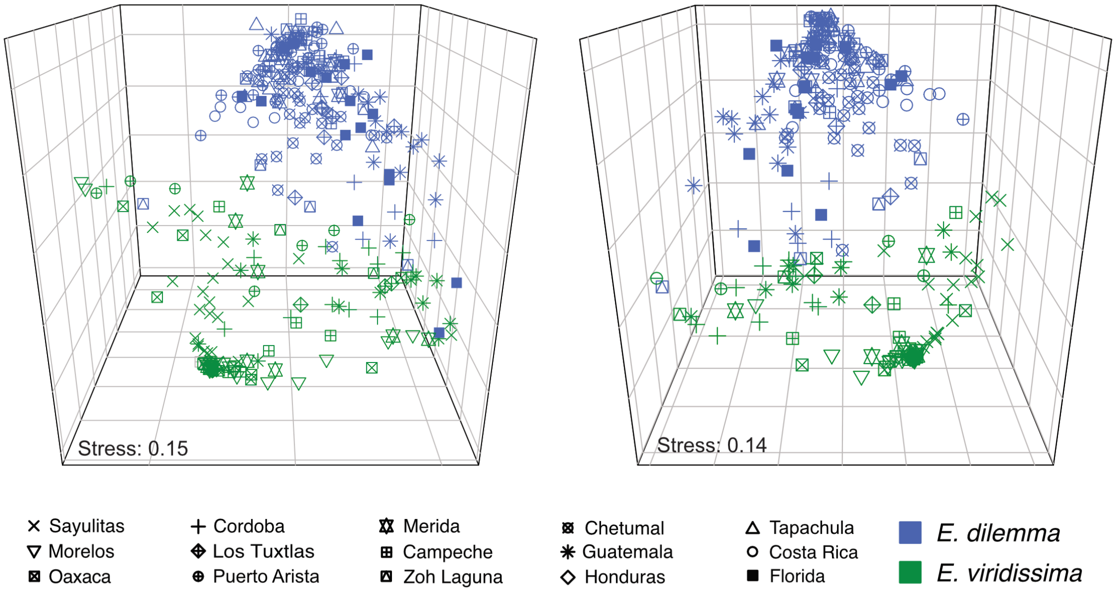

Supplementary Figure 6 Perfume differentiation of *E. dilemma* (green) and *E. viridissima* (blue). Perfume phenotypes clustered species in nMDS analyses based on all perfume compounds (left) and the 40 most abundant compounds (right).

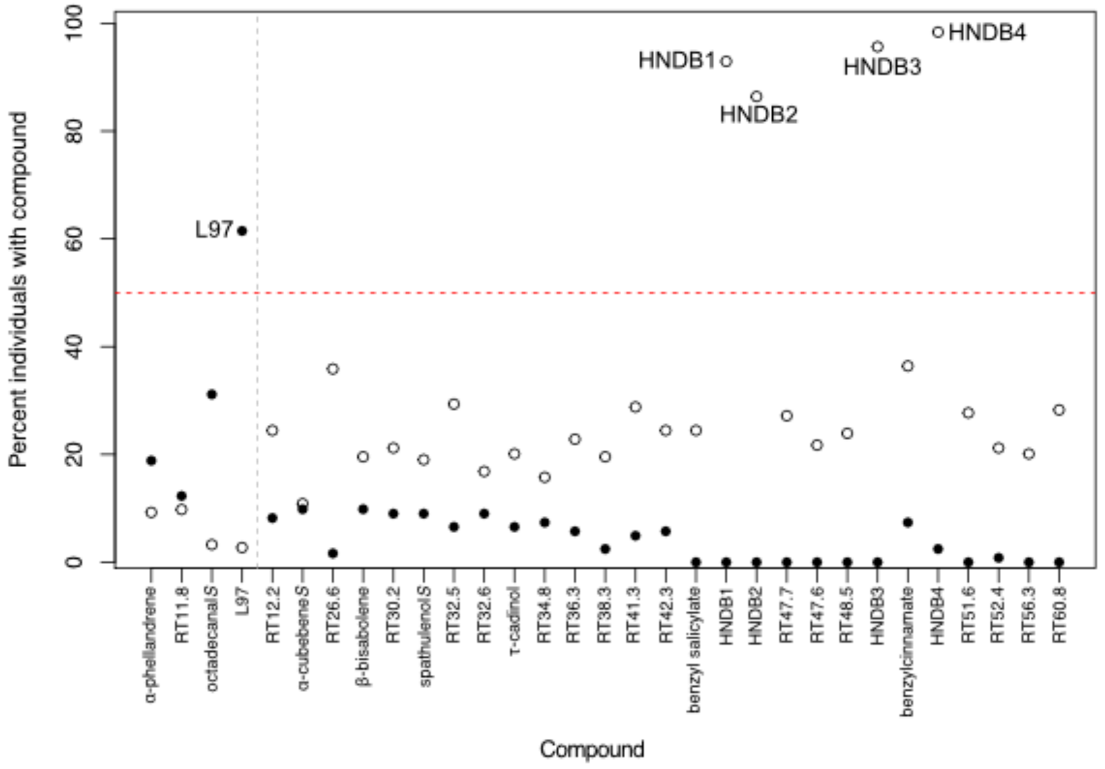

Supplementary Figure 7 Compounds of species-specific high abundance in *E. dilemma* (white) and *E. viridissima* (black). Compounds present in perfumes of >10% of analyzed *E. viridissima* and <10% of analyzed *E. dilemma* individuals (left of grey dotted line) and the reciprocal comparison (right of the grey dotted line) revealed five compounds present in >50% of individuals of one lineage (above red dotted line) and <10% in the other. These are L97

of high abundance in *E. viridissima* and the four HNDB stereoisomers of high abundance in *E. dilemma*. An *S* appended to a compound name indicates compounds similar to the indicated.

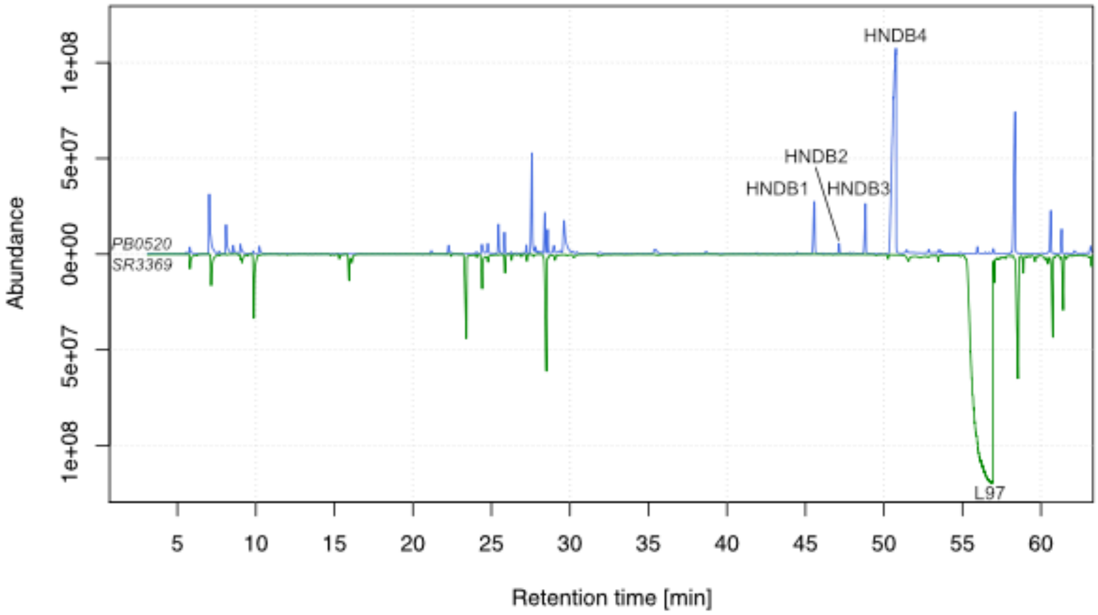

Supplementary Figure 8 Example chromatograms for *E. dilemma* (blue) and *E. viridissima* (green) perfumes highlighting the major compounds. Peaks in the chromatogram represent single compounds present in the perfume of one individual of each lineage (PB0520 and SR3369). The higher a peak and the larger the area under its curve in relation to all other peaks the higher its relative abundance.

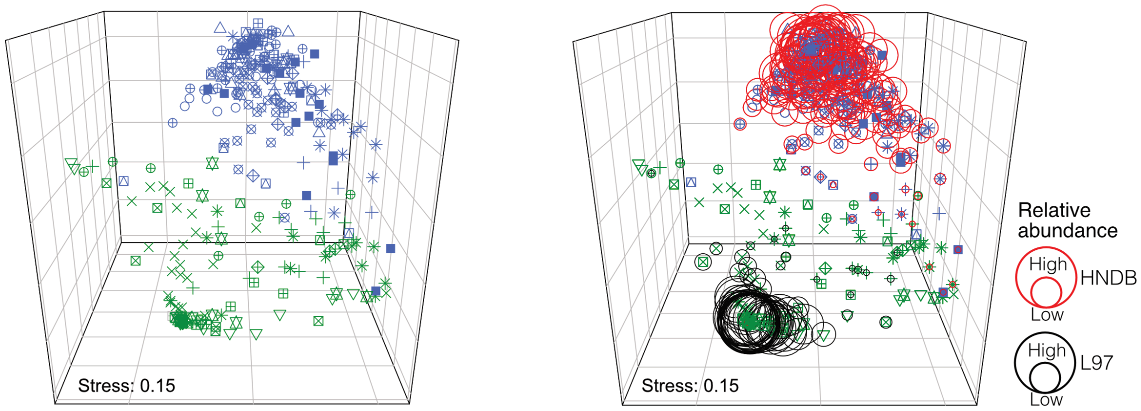

Supplementary Figure 9 Relative abundance of major compounds differentiates perfumes between species. Species-specific clustering of *E. dilemma* (blue) and *E. viridissima* (green) males correlates with high relative abundance of the respective major perfume compounds HNDB (red) and L97 (black).

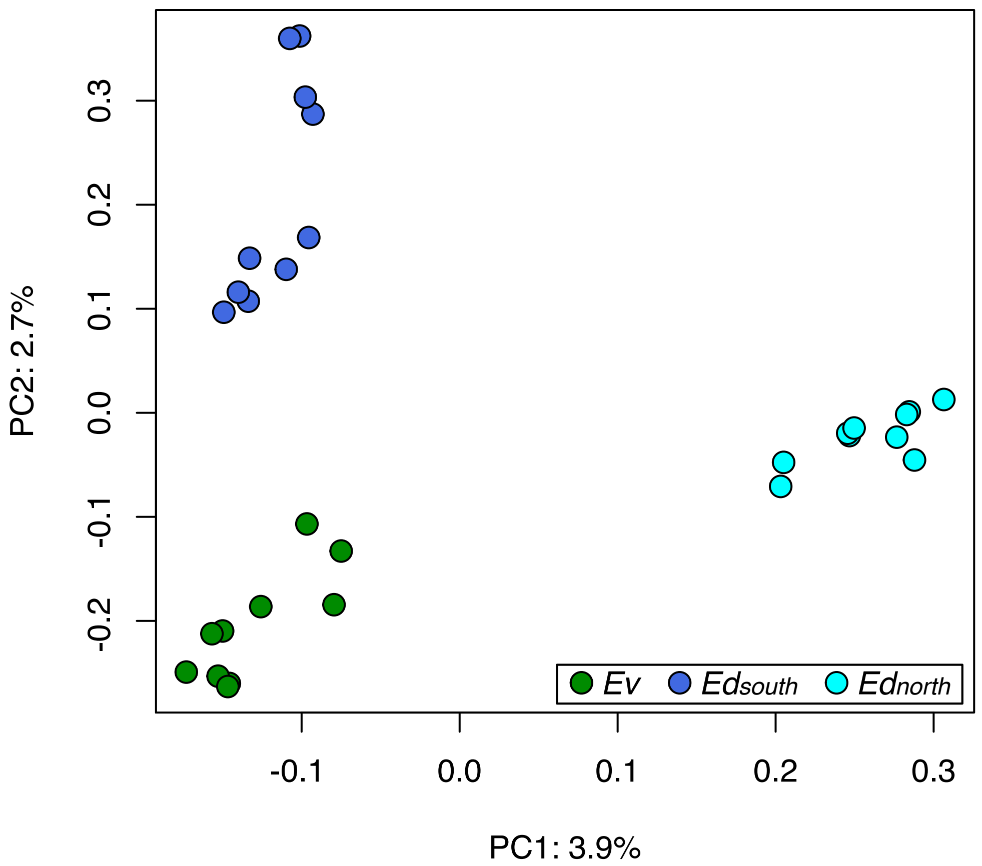

Supplementary Figure 10 PCA of whole-genome re-sequenced individuals and population relationships. *Ev*: *E. viridissima* (green); Ed_{south}: southern *E. dilemma* population (dark blue); Ed_{north}: northern *E. dilemma* population (light blue).

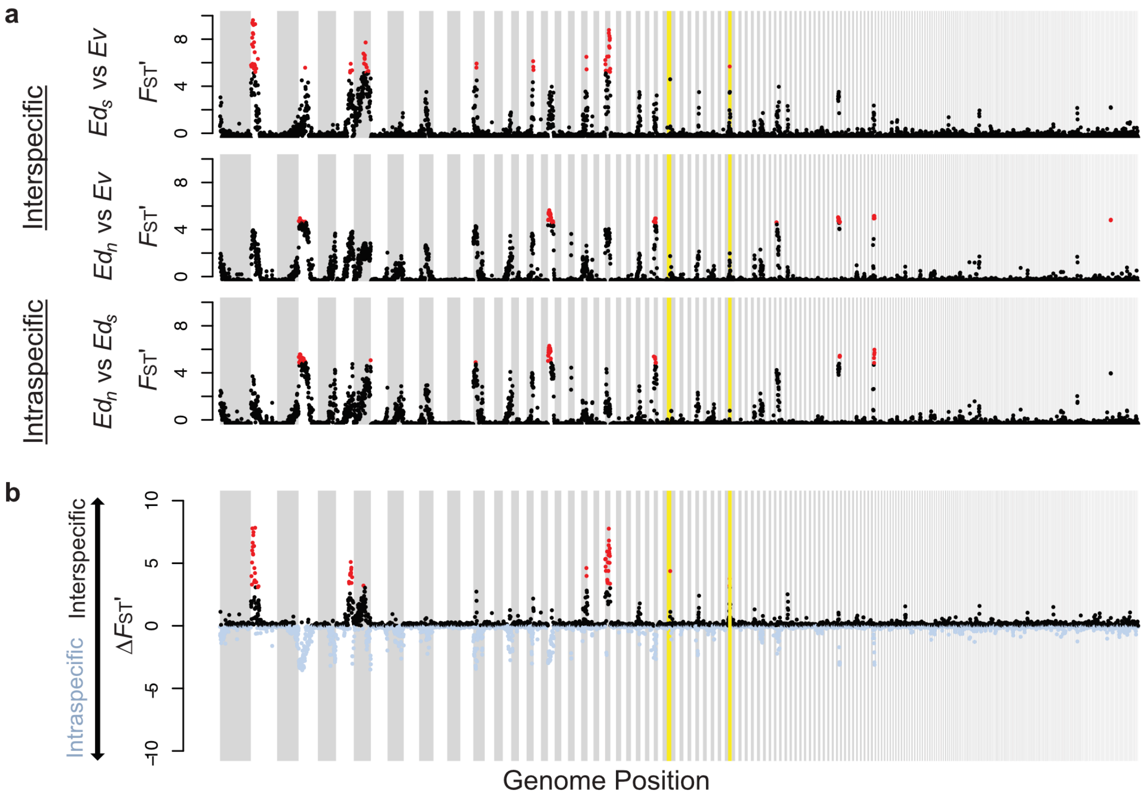

Supplementary Figure 11 Whole-genome differentiation. (**a**) Pairwise comparisons between *Ed_{north}*, *Ed_{south}*, and *Ev* show high heterogeneity of genetic differentiation (F_{ST}') throughout the genome. High-F_{ST}' windows (red; >99th percentile) clustered into peaks that were mostly shared between lineages, indicating a similar genomic landscape across lineages. (**b**) Seven regions of the genome revealed higher interspecific (black) than intraspecific (blue) differentiation (ΔF_{ST} >99th percentile red). The only two ΔF_{ST}' outlier regions identified under strong divergent selection are highlighted in yellow (see main text). *Ed_n*: *Ed_{north}*, *Ed_s*: *Ed_{south}*.

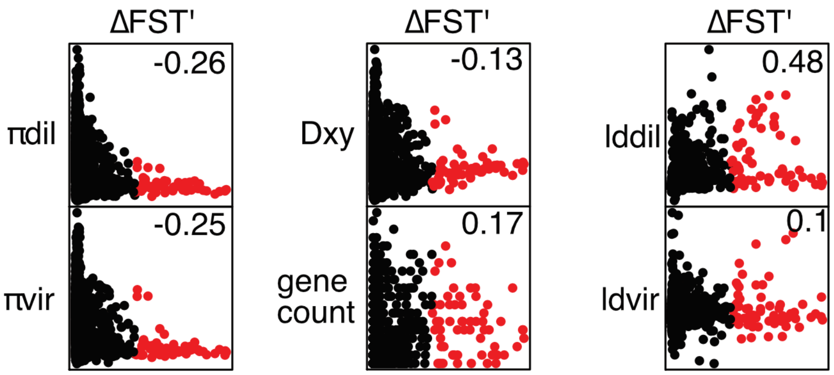

Supplementary Figure 12 Genome-wide correlations between ΔF_{ST} and population genetic parameters. Values inside the plots indicate Pearson's r. All correlations are significant with p = 0. ld: linkage disequilibrium, dil: *E. dilemma*, vir: *E. viridissima*. Outlier windows highlighted in red.

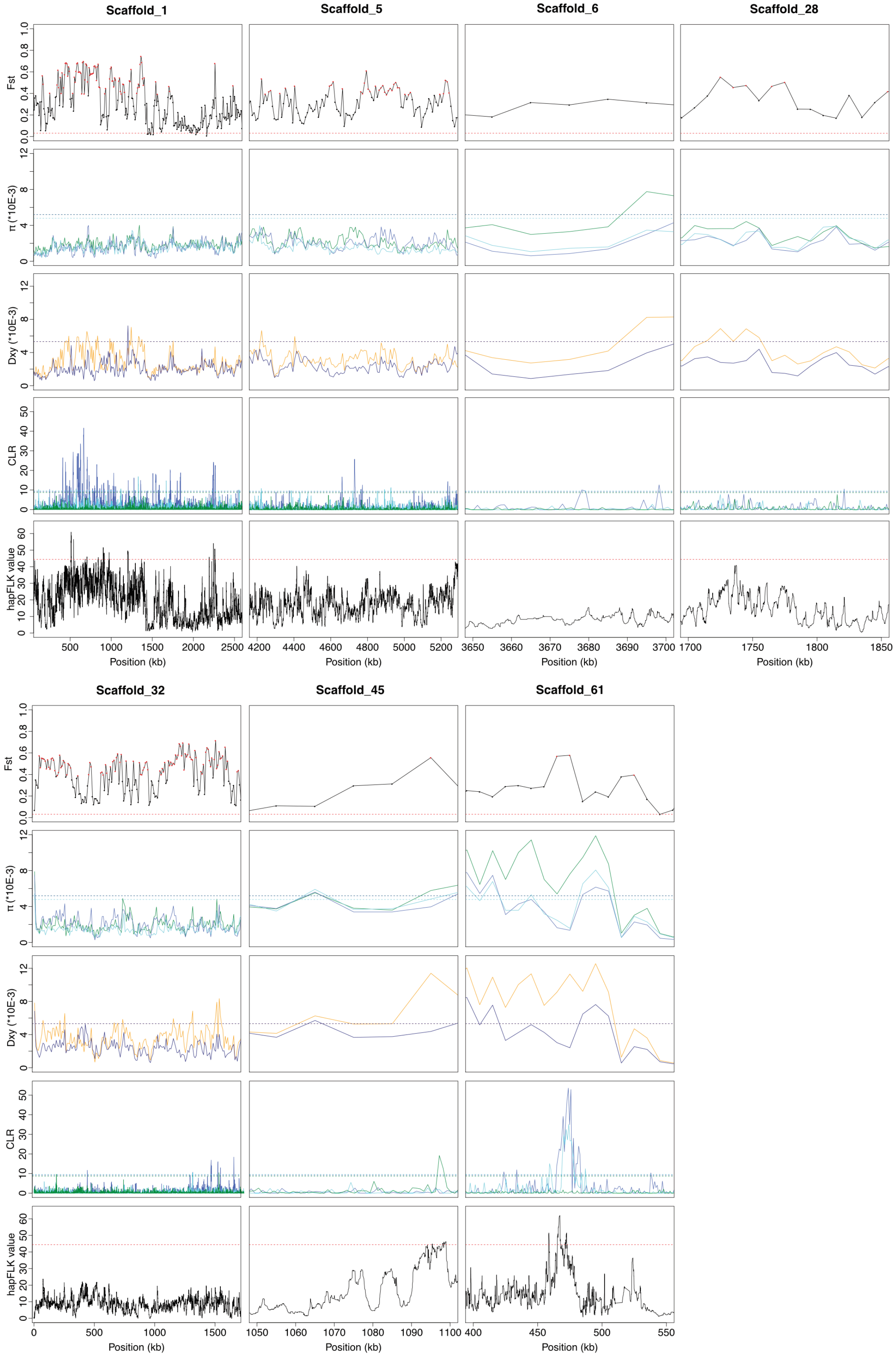

Scaffold_1
Scaffold_5
Scaffold_6
Scaffold_28
Scaffold_32
Scaffold_45
Scaffold_61
Fst
π (*10E-3)
Dxy (*10E-3)
CLR
hapFLK value
Position (kb)

Supplementary Figure 13 Selection analysis in eight outlier peaks with elevated divergence (ΔF_{ST}) between *E. dilemma* and *E. viridissima*. Interspecific F_{ST} (first row), nucleotide diversity (π, second row), absolute divergence (D_{xy}, third row), SweeD CLR score (fourth row), and hapFLK value (fifth row) are shown. F_{ST} (first row): Red points indicate 99 percentile ΔF_{ST} outlier windows (50 kb), red dotted line highlights genome-wide mean interspecific F_{ST}. π (second row): dark blue: Ed_{south}, cyan: Ed_{north}, green: *Ev*, dotted lines: genome-wide mean π for each lineage. D_{xy} (third row): yellow (*E. dilemma* vs. *E. viridissima*), blue (Ed_{north} vs. Ed_{south}), dotted lines: genome wide mean D_{xy}. Sweed CLR (fourth row): dark blue: Ed_{south}, cyan: Ed_{north}, green: *Ev*. hapFLK value (fifth row): red dotted line marks significance threshold, indicating a selective sweep. Elevated F_{ST} in combination with high interspecific differential in π, elevated D_{xy} between *E. dilemma* and *E. viridissima*, and lineage specific CLR outliers as well as significant hapFLK scores were observed only for the outlier region on scaffold_61 harboring *Or41* and the outlier region on scaffold_45 also harboring OR genes (Supplementary Figure 14).

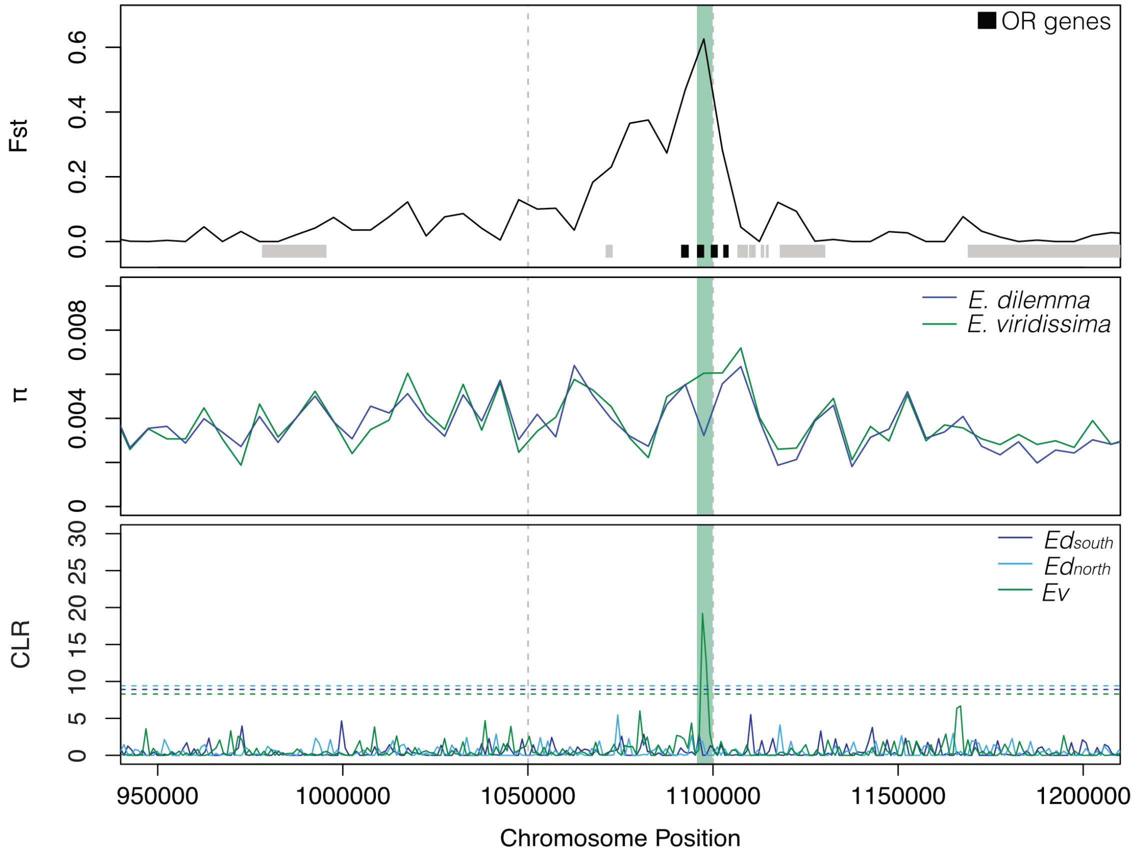

Supplementary Figure 14 *E. viridissima*-specific selective sweep on scaffold_45. The only *E. viridissima*-specific selective sweep identified was located within an F_{ST} outlier window (vertical grey dashed lines) overlapping with a tandem array containing 4 OR genes on scaffold_45. Significant composite likelihood ratios (CLR, bottom) in *Ev* (green) but not Ed_{north} (light blue) and Ed_{south} (dark blue) indicate a selective sweep (highlighted by green shaded region). Horizontal dashed lines in the CLR panel indicate significance threshold for each lineage as indicated in the respective colors.

	40	43	44	56	70	80	86	99	118	129	131	162	167	217	275	305	317	403	407
E. flammea	GTT	GCC	GGT	GAC	ATC	GTC	GTT	ACA	CTG	ATT	TTT	CCT	ATA	CAA	ATC	ATC	CAA	TCA	ATG
E. imperialis	GTT	GCC	GGT	GAC	ATC	GTC	GTT	ACA	CTG	ATT	TTT	CCT	ATA	CAA	ATC	ATC	CAA	TCG	ATG
E. viridissima	GTT	GCC	GGT	GAC	ATC	GTC	GTT	ACA	CTG	ATT	TTT	CCT	ATA	CAT	ATC	ATC	CAA	TCG	ATG
E. dilemma	CTT	GTC	GGC	GGC	CTC	ATC	ATT	ATA	GTG	GTT	GTT	CTT	GTA	GAT	GTC	GTC	CAG	ACG	ACG

Supplementary Figure 15 Fixed substitutions in *Or41* between *E. dilemma* and *E. viridissima* with respect to two outgroup species *E. flammea* and *E. imperialis*. Substitution sites are highlighted in bold. Derived substitutions are indicated in red if non-synonymous and blue if synonymous. *E. imperialis* and *E. flammea Or41* sequences were taken from ref. 1. Numbers indicate the position of the amino acid corresponding to the nucleotide base triplet carrying a substitution.

Supplementary Tables

Supplementary Table 1 Sampling sites

Bait	Latitude	Longitude	IDs	Near	Country	Sampled
crB04	9.6547333	-85.0739333	PB0195-PB0219	Montezuma	Costa Rica	4/20/15
mxB01	18.5844833	-95.0733833	PB0413-PB0435	Los Tuxtlas	Mexico	10/3/15
mxB02	18.5873333	-95.07695	PB0436-PB0457	Los Tuxtlas	Mexico	10/4/15
mxB05	14.8885667	-92.2174	PB0471-PB0517	Tapachula	Mexico	10/8/15
mxB06	15.9339	-93.8113167	PB0518-PB0558	Puerto Arista	Mexico	10/9/15
mxB07	16.8752	-93.4150333	PB0559	On the road	Mexico	10/9/15
mxB08	18.9155333	-96.9823667	PB0560-PB0588	Córdoba	Mexico	10/10/15
mxB09	19.5161	-96.9404333	PB0589-PB0623	Xalapa	Mexico	10/11/15
mxB12	20.89065	-105.4127	PB0675-PB0692	Sayulita	Mexico	10/22/15
ho01	15.5357	-88.3235	PB0693-PB0708	Cusuco	Honduras	Jun/Jul-12
mxB13	15.6711111	-96.5375	SR3369-SR3393	San Augustinillo	Mexico	2/7/16
mxB15	20.7889167	-89.5905333	PB0709-PB0769	Merida	Mexico	5/25/16
mxB16	18.8662667	-88.24675	PB0770-PB0800	Chetumal	Mexico	5/26/16
mxB17	18.6225167	-89.3808167	PB0801-PB0850	Zoh-Laguna	Mexico	5/27/16
mxB18	19.9457667	-90.3739833	PB0851-PB0922	Campeche	Mexico	5/28/16
mxB20	18.9611167	-99.1091167	PB0949-PB0980	Tepoztlan	Mexico	6/4/16
gtB01	16.30504	-89.409445	CLY002-CLY025	Poptún	Guatemala	Apr-16
gtB02	14.61739	-91.521749	CLY026-CLY059	Zapotitlán	Guatemala	9/4/16
usB01	26.228152	-80.186781	cd137-cd140, SR2202-SR2292	Fern Forest	USA	2012-2014

Supplementary Table 2 Pairwise F_{ST} between the three genetic lineages Ed_{south}, Ed_{north}, and Ev. F_{ST} values are shown in the upper right and the number of SNPs used for F_{ST} estimates on the lower left.

	Ed_{south}	Ev	Ed_{north}
Ed_{south}		0.04	0.09
Ev	4135		0.10
Ed_{north}	4128	4134	

Supplementary Table 3 Pairwise F_{ST} between subpopulations of the three genetic lineages.

		Ed_{south}			*Ed_{north}*				*Ev*								
		**TA**	**HO**	**PA**	**ME**	**CA**	**CH**	**ZO**	**PA**	**LO**	**CO**	**GU**	**ME**	**CA**	**SA**	**MO**	**OA**
Ed south	**CR**	0.0 6	0.06	0.09	0.14	0.14	0.15	0.15	0.12	0.13	0.10	0.12	0.13	0.11	0.11	0.12	0.13
	**TA**		0.05	0.05	0.11	0.11	0.11	0.11	0.07	0.10	0.06	0.08	0.08	0.08	0.07	0.08	0.09
	**HO**			0.09	0.13	0.13	0.14	0.14	0.09	0.12	0.09	0.10	0.12	0.11	0.10	0.11	0.12
	**PA**				0.14	0.14	0.14	0.15	0.09	0.12	0.09	0.11	0.12	0.12	0.10	0.11	0.12
Ed north	**ME**					0.07	0.08	0.08	0.15	0.17	0.14	0.16	0.16	0.15	0.15	0.17	0.17
	**CA**						0.07	0.08	0.14	0.17	0.14	0.14	0.15	0.16	0.16	0.16	0.16
	**CH**							0.07	0.16	0.18	0.15	0.16	0.16	0.15	0.16	0.17	0.18
	**ZO**								0.15	0.17	0.14	0.15	0.15	0.16	0.16	0.18	0.18
Ev	**PA**									0.10	0.06	0.09	0.10	0.11	0.07	0.09	0.09
	**LO**										0.08	0.08	0.13	0.11	0.08	0.08	0.08
	**CO**											0.08	0.08	0.08	0.05	0.07	0.07
	**GU**												0.10	0.09	0.09	0.08	0.08
	**ME**													0.10	0.09	0.12	0.12
	**CA**														0.09	0.10	0.11
	**SA**															0.07	0.07
	**MO**																0.06

TA: Tapachula, HO: Honduras, PA: Puerto Arista, ME: Merida, CA: Campeche, CH: Chetumal, ZO: Zoh Laguna, GU: Guatemala, SA: Sayulitas, MO: Morelos, OA: Oaxaca.

Supplementary Table 4 f_4-test for treeness between sympatric and allopatric individuals of *E. dilemma* and *E. viridissima*.

**Test**	f_4	**Std. error**	**Z-score**	**p-value**
$f_4(Ed_{allo},Ed_{sym};Ev_{allo},Ev_{sym})$	0.00128186	0.00047396	2.70458	0.006841

Supplementary Table 5 AIC comparison for demographic models

Model	Bifurcation pattern	log likelihood	Number of parameters	AIC	ΔAIC	AIC weights
migration222	[Ednorth-Edsouth,Edsouth-Ev]	-5057.871	8	10131.74	0	1
migration1222	[Ednorth-Edsouth,Edsouth-Ev]	-5080.086	9	10178.17	46.4	0
migration222	[Ednorth-Ev,Ev-Edsouth]	-5141.289	8	10298.58	100	0

All models up to a ΔAIC of 100 are shown. Model description indicates migration between lineages after first split (1) and second split (2). The repetition of the number indicates number of different vertices (max 1 for 1; max 3 for 2). Bifurcation pattern indicates the lineages resulting from each of the two splits.

Supplementary Table 6 Compounds of highest prevalence in the dataset. Abundance of the 40 most prevalent compounds in the dataset as well as in subsets of *E. dilemma* (Edil) and *E. viridissima* (Evir) are indicated. The last column indicates compounds that contributed more than 1% to perfume differences between species based on a SIMPER analysis (Supplementary Table 8). For compounds that could not be identified the retention time is indicated.

Compound	Individuals total	Prevalence total [%]	Individuals Edil	Prevalence Edil [%]	Individuals Evir	Prevalence Evir [%]	SIMPER >1%
cis-β-ocimene	268	87.58	156	84.78	112	91.80	x
α-pinene	252	82.35	142	77.17	110	90.16	x
sabinene	247	80.72	143	77.72	104	85.25	x
β-pinene	234	76.47	136	73.91	98	80.33	
isoelemicin	206	67.32	142	77.17	64	52.46	x
β-caryophyllene	203	66.34	124	67.39	79	64.75	x
(-)-terpinen-4-ol	197	64.38	112	60.87	85	69.67	x
HNDB4	184	60.13	181	98.37	3	2.46	x
α-caryophyllene	178	58.17	109	59.24	69	56.56	
HNDB3	176	57.52	176	95.65	0	0	x
HNDB1	171	55.88	171	92.93	0	0	x
eugenol	161	52.61	105	57.07	56	45.90	x
HNDB2	159	51.96	159	86.41	0	0	
cis-β-terpineol	151	49.35	90	48.91	61	50.00	
trans-β-ocimene	148	48.37	89	48.37	59	48.36	
germacreneD	146	47.71	84	45.65	62	50.82	x
linalool	144	47.06	93	50.54	51	41.80	
similar to elemicin	143	46.73	103	55.98	40	32.79	
τ-terpinen	141	46.08	88	47.83	53	43.44	
terpinolen	138	45.10	73	39.67	65	53.28	
1,8-cineol	137	44.77	77	41.85	60	49.18	
RT53.4	135	44.12	77	41.85	58	47.54	
D-limonene	128	41.83	77	41.85	51	41.80	x
1-Isopropyl-4-methylbicyclo[3.1.0]hex-2-ene	125	40.85	80	43.48	45	36.89	
α-terpinene	118	38.56	72	39.13	46	37.70	
α-copaene	117	38.24	73	39.67	44	36.07	
tau-elemene	115	37.58	66	35.87	49	40.16	
similar to β-bourbonene	112	36.60	65	35.33	47	38.52	
similar to β-cubebene	111	36.27	72	39.13	39	31.97	
benzyl benzoate	106	34.64	93	50.54	13	10.66	x
RT28.7	101	33.01	66	35.87	35	28.69	x
o-cymene	96	31.37	49	26.63	47	38.52	
RT26.6	96	31.37	52	28.26	44	36.07	
similar to τ-elemene	96	31.37	57	30.98	39	31.97	
similar to thujopsene	95	31.05	58	31.52	37	30.33	
L97	80	26.14	5	2.717	75	61.48	x
β-elemene	77	25.16	51	27.72	26	21.31	
benzylcinnamate	76	24.84	67	36.41	9	7.38	x
1-terpinen-4-ol	74	24.18	50	27.17	24	19.67	
RT27.6	72	23.53	28	15.22	44	36.07	

HNDB: 2-hydroxy-6-nona-1,3-dienyl-benzaldehyde; L97: fatty acid lactone derivative of linolenic acid; RT: retention time.

Supplementary Table 7 Mean relative abundance of compounds in relation to overall perfume composition. All compounds with more than 1% relative abundance in either species are presented for *E. dilemma* and *E. viridissima*. The last column indicates compounds that contributed more than 1% to perfume differences between species based on a SIMPER analysis (Supplementary Table 8). Orange shaded compounds are the major compounds including three stereoisomers of HNDB.

**Compound**	***E. dilemma***	***E. viridissima***	**SIMPER>1%**
L97	0.04	37.28	x
HNDB4	48.67	0.02	x
HNDB3	2.74	0.00	x
HNDB1	2.73	0.00	x
cis-β-ocimene	5.94	8.81	x
benzyl benzoate	5.90	0.07	x
eugenol	5.17	8.27	x
benzylcinnamate	2.96	0.41	x
β-caryophyllene	2.62	3.24	x
isoelemicin	2.32	3.35	x
sabinene	1.36	2.24	x
germacreneD	1.27	3.66	x
D-limonene	0.92	1.95	x
similar to β-cubebene	0.78	1.05	
similar to elemicin	0.75	1.06	
(-)-terpinen-4-ol	0.69	1.97	x
α-pinene	0.60	3.05	x
RT28.7	0.53	2.79	x
1,8-cineol	0.35	1.13	

HNDB: 2-hydroxy-6-nona-1,3-dienyl-benzaldehyde; L97: fatty acid lactone derivative of linolenic acid; RT: retention time.

Supplementary Table 8 SIMPER analysis. Compounds are ranked by their contribution to chemical dissimilarity of perfumes between *E. dilemma* and *E. viridissima*. Orange shaded compounds are the major compounds including three stereoisomers of HNDB.

**Compound**	**average contribution to species differences**	**sd**	**Cumulative sum**
HNDB4	0.243252592	0.110495873	0.2622603
L97	0.186377211	0.200634962	0.4632009
eugenol	0.055175308	0.088609215	0.5226876
cis-β-ocimene	0.053654233	0.069318033	0.5805343
benzyl benzoate	0.029565737	0.048428551	0.6124103
isoelemicin	0.023095267	0.049634033	0.6373103
β-caryophyllene	0.021834442	0.033611296	0.6608508
germacreneD	0.021382415	0.042888817	0.6839041
benzylcinnamate	0.016454031	0.040571676	0.7016438
RT28.7	0.015511842	0.03914692	0.7183677
α-pinene	0.015407743	0.027173899	0.7349794
sabinene	0.013851618	0.025709943	0.7499134
HNDB3	0.013712336	0.009376406	0.7646972
HNDB1	0.013629247	0.011643321	0.7793914
D-limonene	0.012982779	0.03795805	0.7933887
(-)-terpinen-4-ol	0.011348614	0.040367243	0.8056241

HNDB: 2-hydroxy-6-nona-1,3-dienyl-benzaldehyde; L97: fatty acid lactone derivative of linolenic acid; RT: retention time.

Supplementary Table 9 Location and size of detected ΔF_{ST} outlier peaks. Location of the start and end of each outlier peak on the respective genomic scaffold (*sensu* ref. 2) are shown.

Scaffold	Start	End
scaffold_1	150,001	2,500,000
scaffold_5	4,200,001	5,250,000
scaffold_6	3,650,001	3,700,000
scaffold_28	1,700,001	1,850,000
scaffold_32	50,001	1,650,000
scaffold_45	1,050,001	1,100,000
scaffold_61	400,001	550,000

Supplementary Table 10 McDonald-Kreitman test for ORs located in the two tandem arrays with selective sweep signatures.

Selective sweep region	OR gene	Synonymous fixed	Non-synonymous fixed	Synonymous polymorphic	Non-synonymous polymorphic	p
scaffold_61	OR108like_1	0	0	0	4	1
scaffold_61	OR109	0	0	9	1	1
scaffold_61	OR110	1	0	9	2	1
scaffold_61	OR177	0	0	7	0	1
scaffold_61	OR178	0	0	4	3	1
scaffold_61	OR45	0	0	0	6	1
scaffold_61	OR45like_1	1	3	1	1	1
scaffold_61	OR46	0	0	2	0	1
scaffold_61	OR47	0	0	2	0	1
scaffold_61	OR48	0	1	3	3	1
scaffold_61	OR49	1	4	2	0	0.14
scaffold_61	OR49like_1	0	0	4	4	1
scaffold_61	OR50	3	10	2	5	1
scaffold_61	OR95	0	0	9	1	1
scaffold_61	OR97	0	0	9	1	1
scaffold_61	OR100	0	0	4	3	1
scaffold_61	OR101	0	0	3	0	1
scaffold_61	OR105	0	0	4	0	1
scaffold_61	OR106	0	0	10	4	1
scaffold_61	OR107	0	0	7	7	1
scaffold_61	OR108	0	0	2	1	1
scaffold_61	OR41	2	17	6	3	0.005
scaffold_61	OR37	4	1	5	3	1
scaffold_61	OR38	1	0	6	4	1
scaffold_61	OR39	1	0	5	4	1
scaffold_61	OR39like_1	0	3	2	10	1
scaffold_61	OR40like_2	0	0	13	9	1
scaffold_61	OR40like_3	0	0	11	10	1
scaffold_61	OR40like_4	0	0	4	7	1
scaffold_61	OR42	0	0	3	2	1
scaffold_61	OR43	0	0	5	8	1
scaffold_61	OR44like_2	0	0	1	11	1
scaffold_61	OR44like_3	1	0	7	3	1
scaffold_45	OR34	0	0	10	2	1
scaffold_45	OR35	4	4	5	4	1
scaffold_45	OR35like_1	0	1	7	1	0.2
scaffold_45	OR36	1	1	2	4	1

Supplementary Table 11 Primers for tiled OR41 sequencing and OR41 cloning. UTR: Untranslated region. Asterisk indicates cloning primers.

Primer	Sequence 5' -> 3'	Product size	Notes
OR41.01fwd	CGCCATGTTTCACAAGAGAATG	846	starts in 5' UTR; ends in exon 2
OR41.01rev	GGTACAGGTTGTTGCACGAG		
OR41.02fwd	TCTCACGCGTATTTCTATG	393	starts in intron 1; ends in exon 3
OR41.02rev	AGTTACATTCCTTTCCCTGTTTA		
OR41.03fwd	GAGATTATGCACCACCCTC	836	starts in exon 2; ends in intron 4
OR41.03rev	ACGGTATTACTTAAAACGTATCG		
OR41.04fwd	GAATTATTGCAAGAATCTGGAG	885	starts in exon 4; ends in 3' UTR
OR41.04rev	ACCGTCATTGTGAGAAATATCA		
OR41.Ed.fwd*	ggccgaattcaacATGCATTTCATGCAGGATA	1256	Includes EcoRI cut site and Cavener sequence (lower case)
OR41.Ev.fwd*	ggccgaattcaacATGCATTTCACGCAGGATA		Includes EcoRI restriction cut site and Cavener sequence (lower case)
OR41.rev*	ggccctcgagTTATGCAGCTT		Includes XhoI restriction cut site (lower case)
Orco.fwd*	ggccgaattcaacATGATGAAGTTCAAGCAAC		Includes EcoRI restriction cut site and Cavener sequence (lower case)
Orco.rev*	ggccctcgagTCACTTCAGCTGCACC		Includes XhoI restriction cut site (lower case)

Supplementary Table 12 Individuals sequenced for *OR41*.

Sample	Location	Genotype
PB0216	crB04	Ed_{south}
PB0473	mxB05	Ed_{south}
PB0496	mxB05	Ed_{south}
PB0526	mxB06	Ed_{south}
PB0541	mxB06	Ed_{south}
PB0545	mxB06	Ed_{south}
PB0561	mxB08	*Ev*
PB0564	mxB08	*Ev*
PB0566	mxB08	Ed_{north}
PB0589	mxB08	Ed_{north}
PB0596	mxB09	*Ev*
PB0601	mxB09	Ed_{north}
PB0618	mxB09	Ed_{north}
PB0682	mxB12	*Ev*
PB0685	mxB12	*Ev*
PB0698	ho01	Ed_{south}
PB0699	ho01	Ed_{south}
PB0700	ho01	Ed_{south}
PB0705	ho01	Ed_{south}
PB0729	mxB15	*Ev*
PB0758	mxB15	*Ev*
PB0760	mxB15	*Ev*
PB0761	mxB15	*Ev*
PB0763	mxB15	Ed_{north}
PB0769	mxB15	Ed_{north}
PB0786	mxB16	Ed_{north}
PB0810	mxB17	Ed_{north}
PB0844	mxB17	Ed_{north}
SR640	gtB01	Ed_{north}
SR656	gtB01	Ed_{south}
SR2292	Costa Rica	Ed_{south}
SR2295	Costa Rica	Ed_{south}
SR2303	Florida	Ed_{south}
SR2342	Nicaragua	Ed_{south}
SR2355	Nicaragua	Ed_{south}
SR2365	Nicaragua	Ed_{south}
SR2432	Florida	Ed_{south}
CD136	usB01	Ed_{south}
CD139	usB01	Ed_{south}
CD156	Florida	Ed_{south}
CD160	Costa Rica	Ed_{south}
CD177	Mexico	Ed_{north}
CD183	Mexico	*Ev*
CD213	Mexico	*Ev*
CD243	Mexico	Ed_{north}
CD250	Mexico	*Ev*
CD269	Mexico	Ed_{north}

Supplementary Table 13 Selection test based on the *OR41* phylogeny. The *OR41* phylogeny was tested with *E. dilemma* as foreground and *E. viridissima*, *E. imperialis*, *E. flammea*, and *Ef. mexicana* as background.

Model	d_N/d_S background	d_N/d_S foreground	log-likelihood
background = foreground	0.3969	0.3969	-2482.280816
background ≠ foreground	0.321	3.6283	-2474.225479

Supplementary Discussion

Our geometric morphometric analysis of 175 tridentate individuals of both lineages including 11 of the 12 tridentate *E. viridissima* individuals from the sympatric distribution range revealed that the mean position of the central mandibular tooth is shifted towards the basal tooth in *E. viridissima* tridentate males but that there is considerable overlap in tooth positions among individuals between the two lineages (Supplementary Figure 5). This includes the sympatric area analyzed by Eltz and colleagues[3]. Our results suggest that tridentate *E. viridissima* cannot be unequivocally distinguished from *E. dilemma* individuals in the field. However, the results indicate that individuals with a central mandibular tooth shifted towards the basal tooth are more likely to belong to *E. viridissima*. It is possible that tooth morphology is a labile trait varying among generations. The samples analyzed in ref. 3 were all sampled before 2009, some of them more than 40 years ago and it might be possible that tooth morphology changed over short time scales. Further, it is possible that tridentate *E. viridissima* individuals are a result of hybridization between the two lineages. The fact that all but three tridentate *E. viridissima* individuals were found in sympatric but not allopatric populations lends support to this hypothesis.

Supplementary References

1. Brand, P. & Ramírez, S. R. The Evolutionary Dynamics of the Odorant Receptor Gene Family in Corbiculate Bees. *Genome Biol Evol* **9,** 2023–2036 (2017).
2. Brand, P. *et al.* The Nuclear and Mitochondrial Genomes of the Facultatively Eusocial Orchid Bee Euglossa dilemma. *G3: Genes, Genomes, Genetics* **7,** 2891–2898 (2017).
3. Eltz, T. *et al.* Characterization of the orchid bee Euglossa viridissima (Apidae: Euglossini) and a novel cryptic sibling species, by morphological, chemical, and genetic characters. *Zoological Journal of the Linnean Society* **163,** 1064–1076 (2011).
