# Supplementary material for: The evolution of sexual signaling is linked to odorant receptor tuning in perfume-collecting orchid bees
Source: Nat Commun. 2020 Jan 13;11:244. doi: 10.1038/s41467-019-14162-6 (PMC6957680; doi:10.1038/s41467-019-14162-6)
Supplement: Supplementary file 3 — Reporting Summary [file 41467_2019_14162_MOESM3_ESM.pdf]

nature research

Corresponding author(s): Philipp Brand

Last updated by author(s): Dec 16, 2019

Reporting Summary

Nature Research wishes to improve the reproducibility of the work that we publish. This form provides structure for consistency and transparency in reporting. For further information on Nature Research policies, see Authors & Referees and the Editorial Policy Checklist .

Statistics

For all statistical analyses, confirm that the following items are present in the figure legend, table legend, main text, or Methods section.

n/a	Confirmed	
☐	☒	The exact sample size (n) for each experimental group/condition, given as a discrete number and unit of measurement
☐	☒	A statement on whether measurements were taken from distinct samples or whether the same sample was measured repeatedly
☐	☒	The statistical test(s) used AND whether they are one- or two-sided<br>*Only common tests should be described solely by name; describe more complex techniques in the Methods section.*
☒	☐	A description of all covariates tested
☐	☒	A description of any assumptions or corrections, such as tests of normality and adjustment for multiple comparisons
☐	☒	A full description of the statistical parameters including central tendency (e.g. means) or other basic estimates (e.g. regression coefficient) AND variation (e.g. standard deviation) or associated estimates of uncertainty (e.g. confidence intervals)
☐	☒	For null hypothesis testing, the test statistic (e.g. F, t, r) with confidence intervals, effect sizes, degrees of freedom and P value noted<br>*Give P values as exact values whenever suitable.*
☒	☐	For Bayesian analysis, information on the choice of priors and Markov chain Monte Carlo settings
☒	☐	For hierarchical and complex designs, identification of the appropriate level for tests and full reporting of outcomes
☐	☒	Estimates of effect sizes (e.g. Cohen's d, Pearson's r), indicating how they were calculated

Our web collection on statistics for biologists contains articles on many of the points above.

Software and code

Policy information about availability of computer code

Data collection	OpenChrom, WinEDR, auto-montage pro, tpsUtil, tpsDig, Mass Hunter GCMS aquisition software, SLiM
Data analysis	R including packages SNPrelate, vegan, Hmisc, ecodist, imputeqc, PopGenome<br>ADMIXTURE, fourpop in treemix, plink, vcftools, GATK, SweeD, hapFLK, Geneious, MEGA, mafft, RaxML, PAML

For manuscripts utilizing custom algorithms or software that are central to the research but not yet described in published literature, software must be made available to editors/reviewers. We strongly encourage code deposition in a community repository (e.g. GitHub). See the Nature Research guidelines for submitting code & software for further information.

Data

Policy information about availability of data

All manuscripts must include a data availability statement. This statement should provide the following information, where applicable:

- Accession codes, unique identifiers, or web links for publicly available datasets
- A list of figures that have associated raw data
- A description of any restrictions on data availability

Raw sequence data are available through NCBI (BioProjects PRJNA529235 [https://www.ncbi.nlm.nih.gov/bioproject/529235] and PRJNA388474 [https://www.ncbi.nlm.nih.gov/bioproject/388474]), GCMS data are available through Dryad (https://doi.org/10.5061/dryad.1g1jwstrf)

Field-specific reporting

Please select the one below that is the best fit for your research. If you are not sure, read the appropriate sections before making your selection.

☐ Life sciences ☐ Behavioural & social sciences ☒ Ecological, evolutionary & environmental sciences

For a reference copy of the document with all sections, see nature.com/documents/nr-reporting-summary-flat.pdf

Ecological, evolutionary & environmental sciences study design

All studies must disclose on these points even when the disclosure is negative.

Study description	Male orchid bees of the species E. dilemma and E. viridissima were collected at 15 sampling sites throughout Central America and genetic (n=232 for GBS sequencing, n=30 for whole-genome resequencing), chemical data (n=384), and morphological data (414) was analyzed. Single-sensillum recording was performed for an array of chemicals with an n=5-10 each.
Research sample	Male orchid bees of the species E. dilemma and E. viridissima were collected at 15 sampling sites throughout Central America, representing the entire geographic ranges of both.
Sampling strategy	20-40 males of each species were collected at each of 15 sampling sites to represent the local and global populations.
Data collection	We sampled males of two orchid bee species Euglossa dilemma and E. viridissima between 2014 and 2016 throughout the entire distribution area of each lineage using chemical baits following and hand nets. All but one co-authors collected males.
Timing and spatial scale	We sampled males of two orchid bee species Euglossa dilemma and E. viridissima between 2014 and 2016 on multiple field trips throughout Central America
Data exclusions	Data were excluded if of low complexity in GBS sampling (less than 5000 SNPs sequenced) and GCMS (less than 10 perfume compounds).
Reproducibility	The empty neuron experiment was performed for two independent receptor constructs without any problems.
Randomization	Individuals were selected to represent species and randomly selected within sampling sites.
Blinding	Does not apply to our experiments. In empty neuron experiments odors were randomly applied unless otherwise noted in single sessions and thus odor identity and concentrations needed to be recorded at the same time.

Did the study involve field work? ☒ Yes ☐ No

Field work, collection and transport

Field conditions: We collected bees from 9am to 12am local time in sunny conditions.

Location:

Latitude Longitude IDs Near Country Sampled
9.6547333 -85.0739333 PB0195-PB0219 Montezuma Costa Rica 4/20/15
18.5844833 -95.0733833 PB0413-PB0435 Los Tuxtlas Mexico 10/3/15
18.5873333 -95.07695 PB0436-PB0457 Los Tuxtlas Mexico 10/4/15
14.8885667 -92.2174 PB0471-PB0517 Tapachula Mexico 10/8/15
15.9339 -93.8113167 PB0518-PB0558 Puerto Arista Mexico 10/9/15
16.8752 -93.4150333 PB0559 On the road Mexico 10/9/15
18.9155333 -96.9823667 PB0560-PB0588 Córdoba Mexico 10/10/15
19.5161 -96.9404333 PB0589-PB0623 Xalapa Mexico 10/11/15
20.89065 -105.4127 PB0675-PB0692 Sayulita Mexico 10/22/15
15.5357 -88.3235 PB0693-PB0708 Cusuco Honduras Jun/Jul-12
15.6711111 -96.5375 SR3369-SR3393 San Augustinillo Mexico 2/7/16
20.7889167 -89.5905333 PB0709-PB0769 Merida Mexico 5/25/16
18.8662667 -88.24675 PB0770-PB0800 Chetumal Mexico 5/26/16
18.6225167 -89.3808167 PB0801-PB0850 Zoh-Laguna Mexico 5/27/16
19.9457667 -90.3739833 PB0851-PB0922 Campeche Mexico 5/28/16
18.9611167 -99.1091167 PB0949-PB0980 Tepoztlan Mexico 6/4/16
16.30504 -89.409445 CLY002-CLY025 Poptún Guatemala Apr-16
14.61739 -91.521749 CLY026-CLY059 Zapotitlán Guatemala 9/4/16
26.228152
-80.186781
cd137-cd140, SR2202-SR2292 Fern Forest USA 2012-2014

Access and import/export	Sampling and export of bees was performed with the necessary permits issued to SR (Costa Rica, permit 050-2013-STNAC by the Ministerio del Ambiente y Energía), IH-D (Mexico, permit SGPA/DGVS/09586/15 by the Secretaría de Medio Ambiente y Recursos Naturales), and CLYO (Guatemala, permit 2756/2016 by the Consejo Nacional de Areas Protegidas). Collections on private land were only done with the permission of local owners.
Disturbance	The study design is minimally invasive. A low number of bees given the population sizes were collected. Only males were collected. Females can reproduce without mating.

Reporting for specific materials, systems and methods

We require information from authors about some types of materials, experimental systems and methods used in many studies. Here, indicate whether each material, system or method listed is relevant to your study. If you are not sure if a list item applies to your research, read the appropriate section before selecting a response.

Materials & experimental systems

n/a	Involved in the study
☒	☐ Antibodies
☒	☐ Eukaryotic cell lines
☒	☐ Palaeontology
☐	☒ Animals and other organisms
☒	☐ Human research participants
☒	☐ Clinical data

Methods

n/a	Involved in the study
☒	☐ ChIP-seq
☒	☐ Flow cytometry
☒	☐ MRI-based neuroimaging

Animals and other organisms

Policy information about studies involving animals; ARRIVE guidelines recommended for reporting animal research

Laboratory animals	*For laboratory animals, report species, strain, sex and age OR state that the study did not involve laboratory animals.*
Wild animals	Male bees of the species E. dilemma and E. viridissima were collected.
Field-collected samples	Male bees of the species E. dilemma and E. viridissima were collected.
Ethics oversight	No ethical approval or guidance is required for work with insects.

Note that full information on the approval of the study protocol must also be provided in the manuscript.
