# Supplementary material for: The evolution of sexual signaling is linked to odorant receptor tuning in perfume-collecting orchid bees
Source: Nat Commun. 2020 Jan 13;11:244. doi: 10.1038/s41467-019-14162-6 (PMC6957680; doi:10.1038/s41467-019-14162-6)
Supplement: Supplementary file 4 — Description of Additional Supplementary Files [file 41467_2019_14162_MOESM4_ESM.pdf]

Description of Additional Supplementary Files

File Name: Supplementary Data 1

Description: **Individual samples.** Use of each individual in genotyping-by-sequencing (GBS), perfume phenotyping, whole-genome sequencing (WGS), and mandible morphometry is indicated. * Sampling sites correspond to Fig. 1a in the main text. ** Only males included in the morphometric analysis are indicated. *** All males with two mandibular teeth in the dataset are indicated. All others had three teeth. 1R: indicates individuals for which the right mandible was used and mirrored for morphometry. Species status is indicated (Edil: E. dilemma, Evir: E. viridissima).
